# Mycophenolate Mofetil in the Treatment of Steroid-Dependent or Frequently Relapsing Nephrotic Syndrome in Children: A Meta-Analysis

**DOI:** 10.3389/fped.2021.671434

**Published:** 2021-06-15

**Authors:** Xin Xiang, Shi-Yuan Qiu, Mo Wang

**Affiliations:** ^1^Ministry of Education Key Laboratory of Child Development and Disorders, Department of Nephrology, National Clinical Research Center for Child Health and Disorders, China International Science and Technology Cooperation Base of Child development and Critical Disorders, Children's Hospital of Chongqing Medical University, Chongqing, China; ^2^Chongqing Key Laboratory of Pediatrics, Chongqing, China

**Keywords:** mycophenolate mofetil, frequently relapsing nephrotic syndrome, steroid-dependent nephrotic syndrome, children, meta-analysis

## Abstract

**Objectives:** This meta-analysis aims to evaluate the efficacy and safety of the mycophenolate mofetil (MMF) in the treatment of steroid-dependent nephrotic syndrome (SDNS) or frequently relapsing nephrotic syndrome (FRNS) in children.

**Methods:** We searched for the studies especially the randomized controlled trials in PubMed, Cochrane Library, Embase, China National Knowledge Infrastructure, and Wan Fang database. The data were analyzed by Review Manager 5.3 software. We used the GRADE pro-Guideline Development Tool online software to evaluate the quality of evidence.

**Results:** Finally, we identified 620 studies, of which we included five randomized controlled trials and one prospective cohort study with 447 children. The results showed the following: (1) the relapse-free survival rate within 1 year—the MMF group was superior to the levamisole group [ratio difference (RD) = 0.13, 95% CI (0.02, 0.24), *P* = 0.02] but not to the calcineurin inhibitors (CNIs) group [RD = −0.27, 95%CI (−0.40, −0.14), *P* < 0.0001]; (2) the number of relapses within 1 year—the MMF group was less than that in the CNIs and levamisole group [mean difference (MD) = −0.26, 95%CI (−0.45, −0.08), *P* = 0.005]; (3) the cumulative prednisone dosage—the MMF group was lower than that in the control group [standardized mean difference (SMD) = −0.32, 95%CI (−0.53, −0.11), *P* = 0.003]; (4) incidence of adverse reactions—there was no significant difference between the MMF group and the control group [RD = 0.02, 95%CI (−0.04, 0.09), *P* = 0.46].

**Conclusion:** The therapy of mycophenolate mofetil in the treatment of SDNS or FRNS in children has a certain advantage in reducing the number of relapses and cumulative prednisone dosage within 1 year when compared with the CNIs and levamisole. However, due to the limited quantity and quality of the included studies, the conclusions above need to be confirmed by more high-quality randomized controlled trials.

## Introduction

Nephrotic syndrome (NS) is a common glomerular disease in childhood characterized by proteinuria, hypoproteinemia, hyperlipidemia, and edema. The pathogenesis of the disease has not been fully elucidated. At present, it is mainly related to immune imbalance (1), systemic circulatory factors (2), and abnormal podocyte genetic mutations (3). Ninety percent of cases of nephrotic syndrome presenting in childhood are idiopathic (4), and glucocorticoid therapy has been considered a first-line treatment for PNS in children since the 1950s. According to the response to steroid treatment, PNS can be classified as steroid-sensitive nephrotic syndrome (SSNS), steroid-resistant nephrotic syndrome (SRNS), and steroid-dependent nephrotic syndrome (SDNS). SDNS and FRNS are refractory patterns of steroid responsiveness in nephrotic syndrome that account for about 40% of PNS in children whose goal is to choose appropriate second-line immunosuppressants, to induce a response as soon as possible, and to maintain a long-term response. The Kidney Disease: Improving Global Outcomes (KDIGO) guidelines (5) recommend a variety of immunosuppressants for the treatment of SDNS or FRNS in children, including mycophenolate mofetil (MMF), cyclophosphamide, cyclosporine A (CsA), tacrolimus (TAC), rituximab, and the immunomodulator levamisole. However, due to the responsiveness of different patients to different drugs and adverse reactions, making the best plan of pharmacotherapy is still a great challenge for most pediatric nephrologists.

MMF, as a novel immunosuppressant, is a 2-ethyl ester derivative of mycophenolic acid, which is taken off the esterification *in vivo* to form a metabolite with immunosuppressive activity. Mycophenolic acid can selectively act on T and B lymphocytes and the first or second signal in the process of activation to achieve the purpose of immunosuppression. In addition, the inhibitory effect of MMF on other cytokines *in vivo* can also play a role in delaying the progression of the disease. In 1998 (6), American doctors used MMF for the first time in the treatment of adult RNS and achieved good results. Subsequently, the role of MMF in the treatment of kidney disease has received widespread attention. Previous studies have confirmed its positive effect on the treatment of SDNS or FRNS in children. However, most of these studies are based on retrospective analysis, and only a few randomized controlled trials (RCTs) have focused on its efficacy and safety. Therefore, in this study, a meta-analysis was conducted to evaluate the efficacy and safety of MMF in the treatment of SDNS or FRNS in children to provide higher-strength evidence for the usage of MMF.

## Methods

### Study Design

In accordance with the principle of “PICOS,” it was defined as following: (1) P: the children with SDNS or FRNS; (2) I: treated with MMF or other immunosuppressants (TAC, CsA, and levamisole; (3) C: MMF vs. other immunosuppressants (TAC, CsA, and levamisole); (4) O: relapse-free survival rate within 1 year, the number of relapses within 1 year, cumulative prednisone dosage, and incidence of adverse reactions; (5) S: a meta-analysis of RCTs and a prospective cohort study.

### Literature Search

#### Chinese Literature

We searched the China National Knowledge Infrastructure (CNKI) and Wan Fang database with the keywords including “nephrotic syndrome,” “mycophenolate mofetil,” and “children.”

#### English Literature

We used the combination of subject words and free words to search PubMed, Embase, and The Cochrane Library. For example, the free words of “nephrotic syndrome” include “nephrotic syndromes,” “syndrome, nephrotic,” “syndromes, nephrotic,” and “nephrotic.”

#### Search Time

Unlimited–December 2020.

### Study Selection and Data Extraction

#### Inclusion criteria

*Inclusion criteria were as follows: (1)* the prospective studies, especially RCTs, of MMF in the treatment of SDNS or FRNS in children aged from 3 months to 18 years old; (2) the follow-up period of at least 1 year; and (3) the diagnostic criteria refers to the 2012 KDIGO guidelines (7, 8): (a) SDNS, two consecutive relapses during corticosteroid therapy or within 14 days of ceasing therapy; (b) FRNS, >2 relapses within 6 months of initial relapse, or ≥4 relapses in any 12 months. Among them, steroid sensitivity showed that urinary protein turned negative after 4 weeks of treatment with a sufficient amount of prednisone [2 mg/(kg·day) or 60 mg/(m^2^·day)].

#### Exclusion criteria

Exclusion criteria were as follows: (1) all kinds of secondary nephrotic syndrome, such as nephrotic syndrome caused by lupus, hepatitis B virus infection, and antineutrophil-associated glomerulonephritis; (2) retrospective studies, review, meeting, and literature that is not consistent with the purpose of evaluation.

#### Data Extraction

Based on the inclusion and exclusion criteria, independent duplicate data extraction was performed by two reviewers (Xin Xiang and Shi-Yuan Qiu) using a predesigned data collection form, and the results were reviewed by a third investigator (Mo Wang). In this study, a small number of data in the original literature are expressed by the quartile method, which needs to be converted into mean and standard deviation by using Luo (9) and other methods.

### Types of Outcome Measures

#### Primary Outcomes

Relapse-free survival rate within 1 year, the number of relapses within 1 year, and cumulative prednisone dosage.

#### Relapse

Twenty-four-hour urine protein ≥50 mg/kg for 3 consecutive days, urinary protein/creatinine (mg/mg) ≥2.0 in morning urine or morning urine protein changed from negative to positive or (+++)–(++++).

#### Secondary Outcome

Incidence of adverse reactions (mainly considering serious adverse reactions, such as severe infection, agranulocytosis, etc.).

### Statistical Methods

Statistical analysis of the data was carried out by RevMan5.3 software provided by The Cochrane collaboration network. Heterogeneity analysis was carried out in selected trials. When *P* > 0.05 and *I*^2^ <50%, the homogeneity of the study was not significant. A fixed-effect model was used. On the contrary, the random effect model was adopted. Ratio difference (RD) and its 95% confidence interval (95%CI) were used for count data. Mean difference (MD) or standardized mean difference (SMD) and its 95% CI were used for measurement data. If there was obvious heterogeneity in the study, its sensitivity was analyzed and postprocessed. Only descriptive analysis was carried out if it cannot be determined.

### Evidence Quality Assessment

We used the GRADE pro-Guideline Development Tool online software (GRADEpro GDT, Evidence Prime, Hamilton, ON) to evaluate the quality of evidence.

## Results

### Literature Retrieval Results

**Figure 1:**
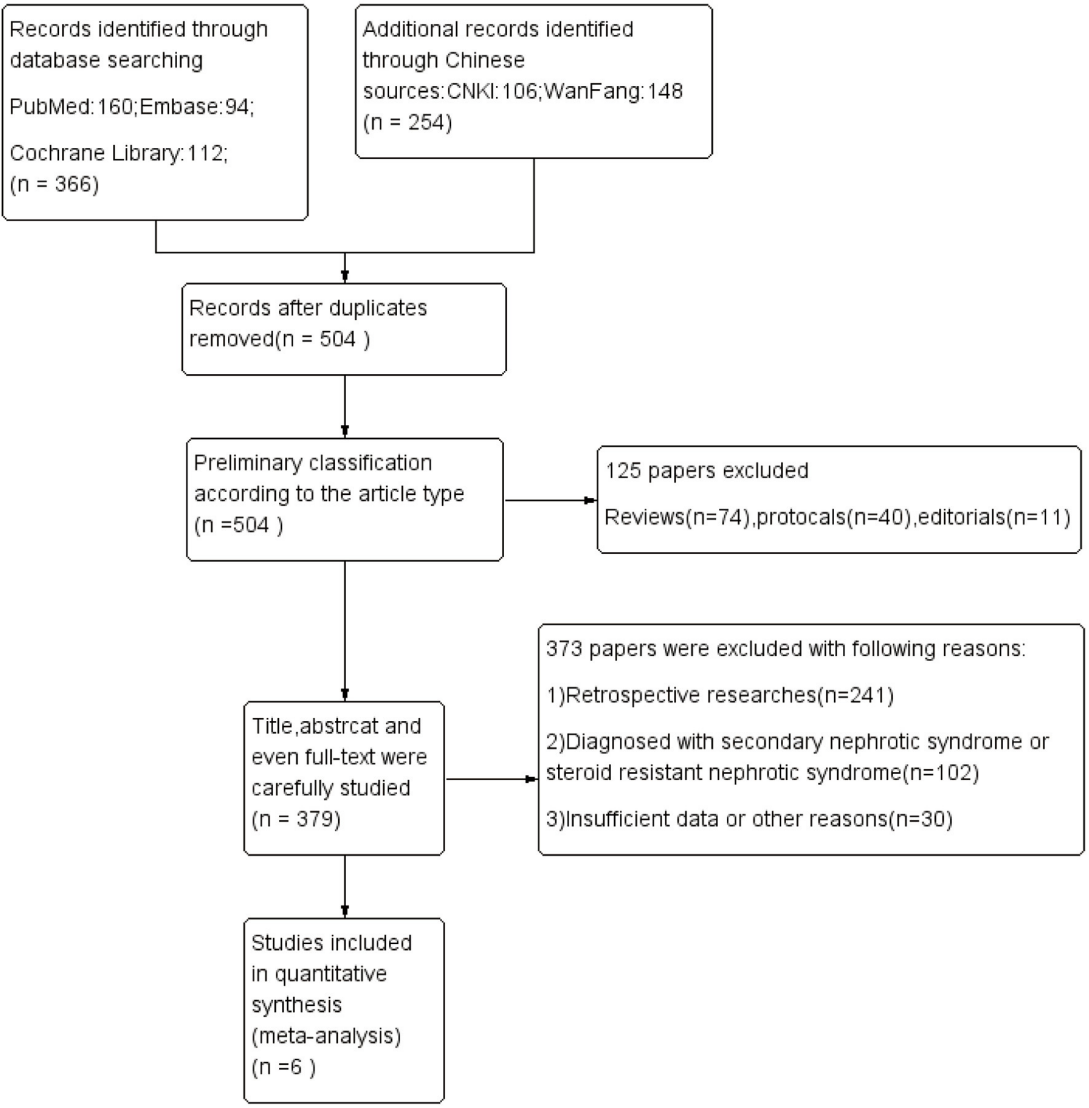
Flow diagram of the study identification.

Six hundred twenty articles were retrieved according to the above method. We excluded the repetitions, reviews, retrospective studies, nonclinical studies, and the studies inconsistent with the purpose of evaluation by reading titles, abstracts, and some of the specific contents of the literature. Finally, a total of six articles were included: one Chinese literature (10) and five English articles (11–15). The total number of subjects analyzed was 447 children. Figure 1 shows the process of literature retrieval. Table 1 shows the characteristics of the included studies.

**Table 1:** Characteristics of the included study.

**Trial**	**Patient number**		**Intervention**		**Renal biopsy**	**Outcome**
	**E^a^**	**C^b^**	**E**	**C**		
Sinha (14)	76	73	MMF 750–1,000 mg/m^2^/d, 1 year	Levamisole 22.5 mg/kg/qod,1 year	No	1^c^ 2^d^ 3^e^ 4^f^
Basu (15)	56	56	MMF 1,200 mg/m^2^/d,1 year	Levamisole 2.5 mg/kg/qod,1 year	No	1 2 3 4
Dorresteijn (13)	12	12	MMF 1,200 mg/m^2^/d,1 year	CsA 4–5 mg/kg/d,1 year	Yes	1 2 3 4
Gellermann (11)	28	30	MMF, target plasma trough level 1.5–2.5 mg/ml	CsA, target plasma trough level 80–100 ng/ml	Yes	1 4
Wang (12)	34	38	MMF 20–30 mg/kg/d, 1 year	TAC 0.05–0.15 mg/kg/d, 1 year	Yes	1 3 4
Geng (10)	14	18	MMF 20–30 mg/kg/d, 1 year	CsA 3–5 mg/kg/d,1 year	Yes	1 2 4

a *Experiment group*.

b *Control group*.

c *1-year relapse-free survival rate*.

d *The number of relapses within 1 year*.

e *Cumulative glucocorticoid dosage*.

f *Incidence of adverse reactions*.

### Risk of Bias

**Figure 2:**
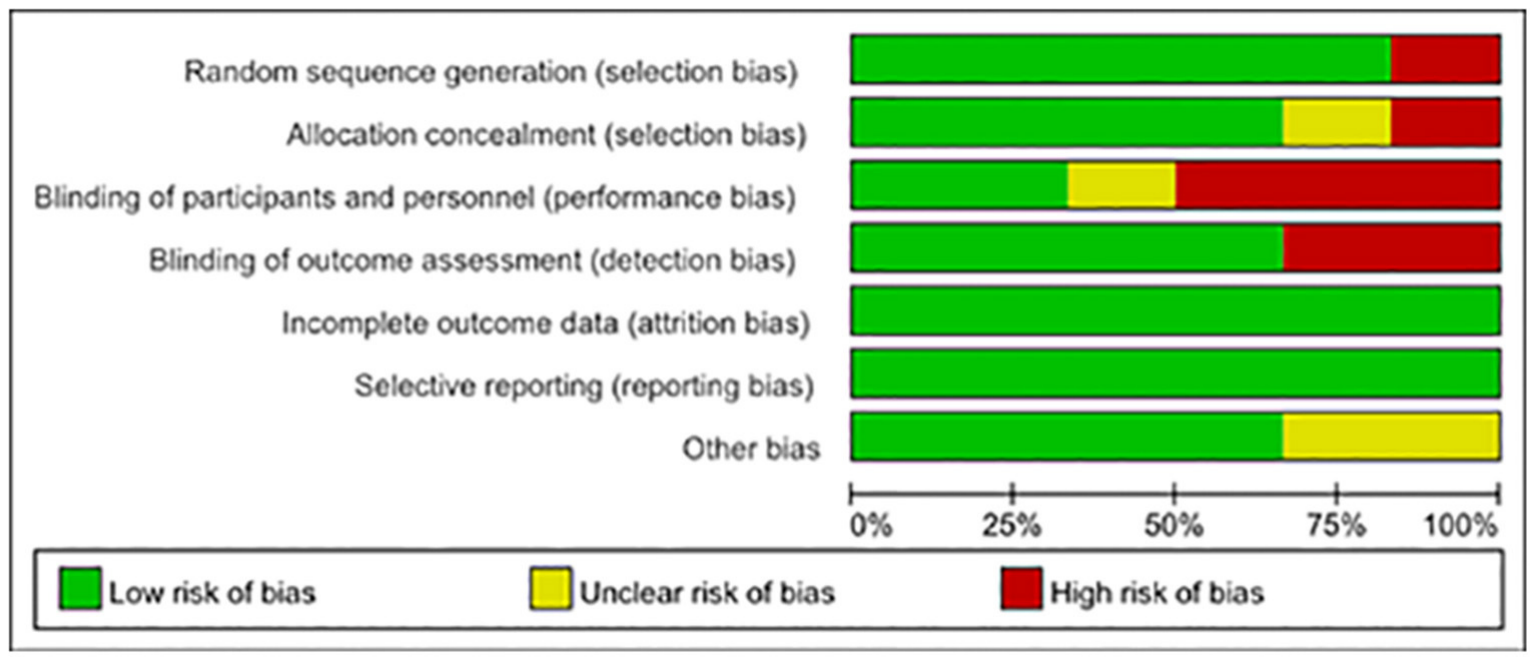
Inclusion bias.

The Cochrane Collaboration (16) was used for assessing the risk of bias. Of the six articles, one was randomly generated by a computer, one was stratified by randomized numbers in Excel table, one was randomized by central computer minimization, and the other two were randomly grouped, whose random allocation method was not indicated. Two of them were hidden in design allocation but without indicating the specific hiding method. One article adopted the blind method, but others did not mention whether to use blind methods. Follow-up after publication was not mentioned in almost all literature. All the studies clearly explained that there was no significant statistical difference in the baseline data between the experimental group and the control group, which means that they were balanced and comparable. Figure 2 shows the authors' judgment about each risk bias item presented as percentages across all included studies.

### 1-Year Relapse-Free Survival Rate

**Figure 3:**
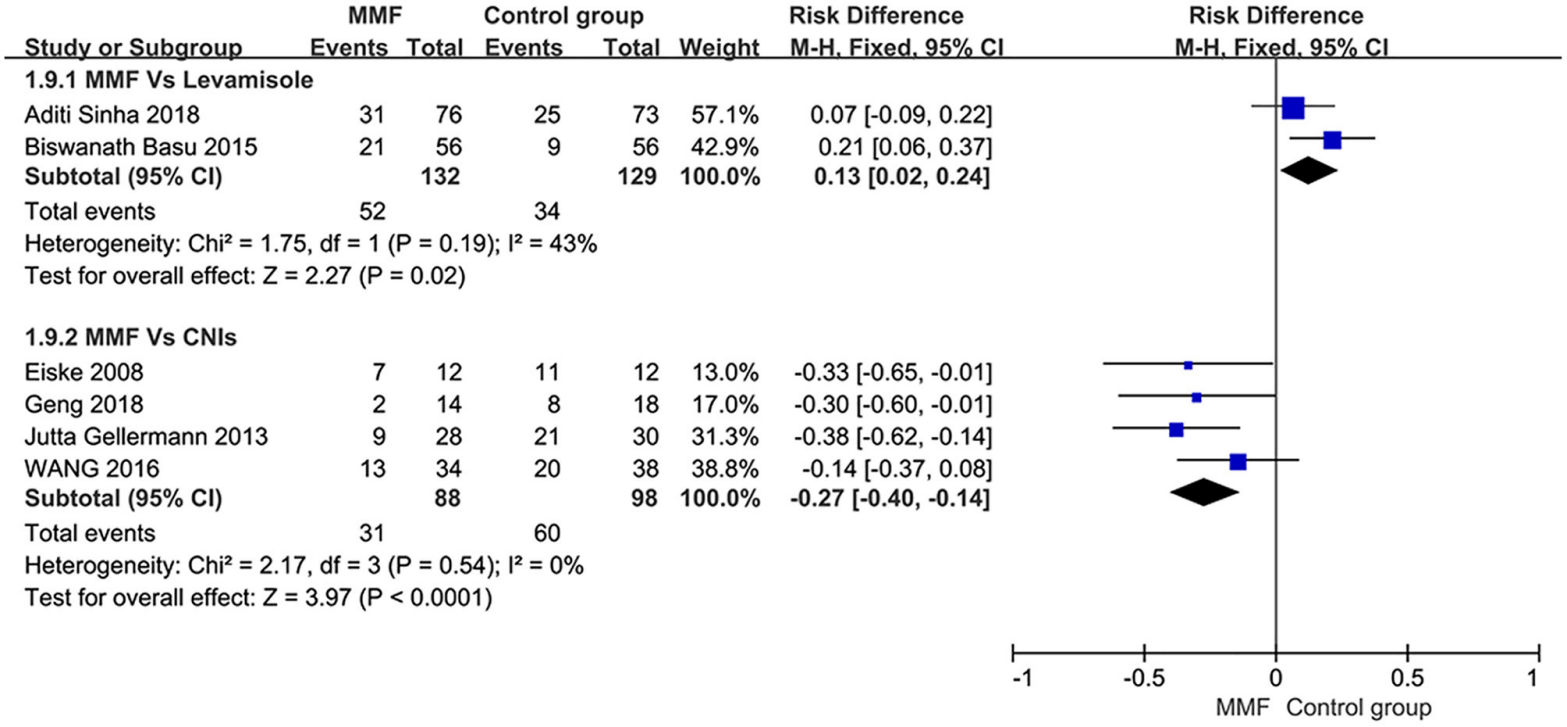
Forest plot showing ratio difference (RD) of the 1-year relapse-free survival rate between the groups.

The definition of relapse of SDNS or FRNS in children is as mentioned above. All of the six articles included in this study reported the relapse-free survival rate within 1 year. When analyzing all the data extracted from the literature, we found that there was significant heterogeneity among the studies. Thus, we conducted the subgroups according to the types of immunosuppressants used in the control group. The results shown in Figure 3 indicate that MMF was superior to levamisole but not to calcineurin inhibitors (CNIs), and the differences were statistically significant.

### The Number of Relapses Within 1 Year

**Figure 4:**
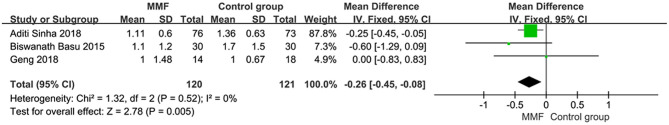
Forest plot showing mean difference (MD) of the number of relapses within 1 year between the groups.

Four articles reported the number of relapses within 1 year. The total number of patients after treatment was analyzed. The results showed that there was heterogeneity among the studies. We have to find the source of heterogeneity by eliminating one by one. After removing the literature of Eiske2008, the heterogeneity was significantly reduced. Re-reading the literature, we found that the renal biopsy results of the subjects included in this study were minimal change diseases (MCDs). We thought it to be the source of heterogeneity, so the literature was deleted. The remaining three articles were analyzed by the fixed-effect model, and the specific results of the meta-analysis were as follows in Figure 4 [MD = −0.26, 95%CI (−0.45, −0.08), *P* = 0.005]. The number of relapses within 1 year was statistically significant between the two groups. Thus, it was considered that the MMF group was superior to the control group in reducing the number of relapses within 1 year.

### Cumulative Prednisone Dosage

**Figure 5:**
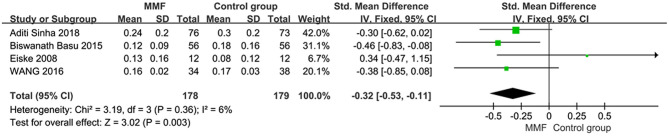
Forest plot showing standardized mean difference (SMD) of the cumulative prednisone dosage between the groups.

Four articles reported the data of cumulative prednisone dosage. There was no obvious heterogeneity among the studies, so the fixed effect model was used for analysis. The specific meta-analysis results are shown in Figure 5. The difference in cumulative hormone usage between children with SDNS or FRNS treated with MMF and the control group was statistically significant [SMD = −0.32, 95%CI (−0.53, −0.11), *P* = 0.003], indicating that MMF is more effective in reducing cumulative prednisone dosage than the CNIs and levamisole.

### Incidence of Adverse Reactions

**Figure 6:**
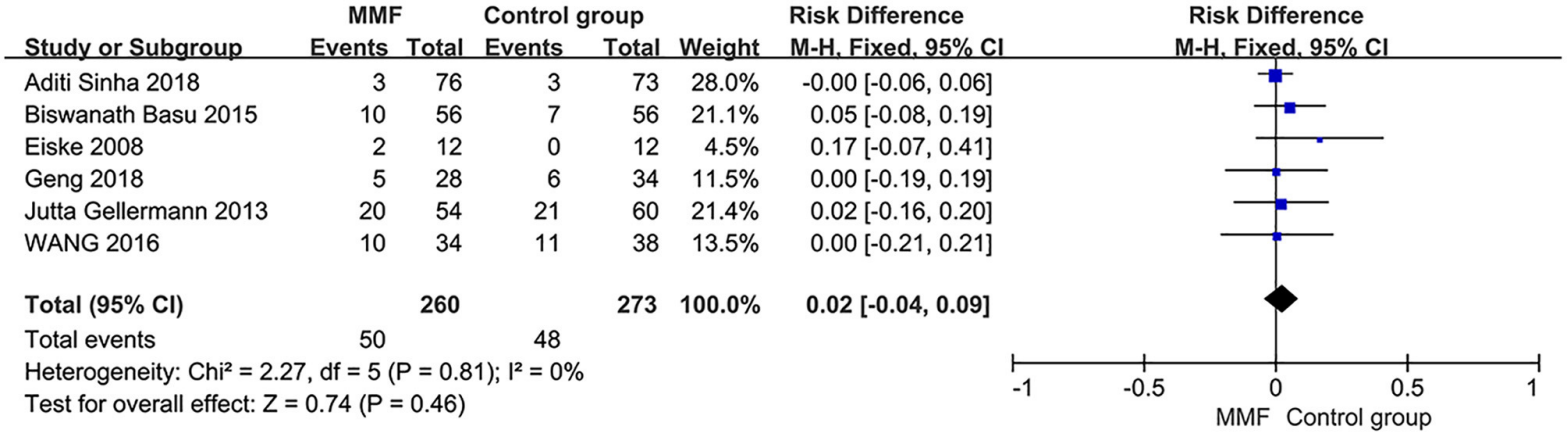
Forest plot showing ratio difference (RD) of the incidence of adverse reactions between the groups.

Heterogeneity analysis was conducted on six articles reporting the incidence of adverse reactions. Then, the fixed-effect model was used for analysis. The results of meta-analysis are shown in Figure 6. There was no significant difference in the incidence of adverse reactions between the MMF group and the control group [RD = 0.02, 95%CI (−0.04, 0.09), *P* = 0.46]. We believe that there is no significant difference in safety between MMF and the control group.

## Discussion

The objective of this study was to evaluate the efficacy and safety of mycophenolate mofetil in the treatment of SDNS or FRNS in children by meta-analysis. Six prospective studies were included, with a total number of 447 children. The results show that when compared with other immunosuppressants (including tacrolimus, cyclosporine A, and levamisole), MMF has some advantages in reducing the number of relapses and cumulative prednisone dosage within 1 year. As for the relapse-free survival, MMF was superior to levamisole but not to CNIs. At the same time, the incidence of adverse reactions did not decrease, which was not consistent with previous research and clinical experience (17, 18). In addition, in order to explore the optimal dose of MMF, we conducted a subgroup analysis of different doses of MMF according to the RCTs included in the study. However, we did not find that the effects of different subgroups on the main outcomes were statistically significant.

We also used GRADE pro-GDT to evaluate the quality of the primary outcomes and secondary outcomes (Table 2). The results suggested that the quality of the evidence in the 1-year relapse-free survival rate was high, while the quality of the evidence for the cumulative prednisone dosage and the incidence of adverse reactions were moderate. However, the certainty of the number of relapses within 1 year was low.

**Table 2:** Evaluation of GRADE pro GDT of MMF or others for the treatment of SDNS or FRNS in children.

**Certainty assessment**							№ **of patients**		**Effect**		**Certainty**	**Importance**
**Outcomes (No of studies)**	**Study design**	**Risk of bias**	**Inconsistency**	**Indirectness**	**Imprecision**	**Other considerations**	**MMF**	**Others**	**Relative (95% CI)**	**Absolute (95% CI)**		
1-year relapse-free survival rate (6)	Random-ized trials (5/6)	Not serio-us	Not serious	Not serious	Not serious	None	80/220 (37.9%)	94/227 (41.4%)	RD−0.02 (-0.12 to 0.05)	-	⊕⊕⊕⊕ **HIGH**	IMPORTANT
The number of relapses within 1 year (3)	Randomized trials	Not serious	Not serious	Serious^a^	Serious^b^	None	120	121	-	MD 0.26 lower (0.45 lower to 0.08 lower)	⊕⊕◯◯ **LOW**	CRITICAL
Cumulative prednisone dosage (4)	Randomized trials (3/4)	Not serio-us	Not serious	Not serious	Serious^b^	None	178	179	-	SMD 0.32 lower (0.53 lower to 0.11 lower)	⊕⊕⊕◯ **MODERATE**	CRITICAL
Incidence of adverse reactions (6)	Randomized trials (5/6)	Not serious	Serious^c^	Not serious	Not serious	None	50/260 (19.2%)	48/273 (17.6%)	RD 0.02 (−0.04 to 0.09)	-	⊕⊕⊕◯ **MODERATE**	IMPORTANT

a *The mean and standard deviation of the article with largest weight are converted from the quartile notation by Luo's methods*.

b *Limited sample size*.

c *Different articles have different definitions of severe and mild adverse reactions*.

*⊕, Evidence quality symbol; ◯, Downgraded one level*.

One of the main outcomes of this study is the incidence of adverse reactions, which are mainly concerned with serious adverse reactions, such as severe infection and agranulocytosis. However, the mild adverse reactions such as rash and medicated fever may be ignored. Almost all kinds of immunosuppressants have varying degrees of side effects. This also has become one of the reasons why it is difficult for us to choose appropriate treatment plans in clinical work. It was reported that the side effects of MMF are cytopenia and diarrhea. Levamisole has an immunomodulatory function that is usually well-tolerated. Its main side effect is elevated liver enzymes. Calcineurin inhibitors have long been used in SDNS or FRNS. Their major side effects are hirsutism, gum hypertrophy, and nephrotoxicity, leading to interstitial kidney fibrosis and chronic kidney disease. Cyclophosphamide is an efficient treatment, but its gonadal toxicity is a major drawback to its use. More recent drugs such as rituximab are very effective but induce an increased risk of opportunistic infection, prolonged neutropenia, and anaphylaxis.

MMF can be used not only in the treatment of PNS but also in other kidney diseases in children. By comparing the different efficacy of MMF combined with steroid and steroid alone in the treatment of 76 children with Henoch–Schoenlein purpura nephritis (HSPN), Lu et al. (19) found that the combination of MMF and steroid was superior to steroid alone in relieving proteinuria. In the treatment of antineutrophil cytoplasmic antibody (ANCA)-associated vasculitis (AAV). Hu et al. (20) found that MMF effectively ameliorates disease activity and considerably improves renal function in patients. Similarly, MMF also plays a significant role in renal transplant patients (21).

Due to the limited number of clinical researches in pediatric population and short application history of MMF, the data included in this study were insufficient. By consulting the Chinese Clinical Trail Registry (ChiCTR) and International Clinical Trails Registry Platform (WHO ICTRP), we found that some clinical projects in line with the theme of our research, including IRCT20130812014333n113, NCT04048161, CTRI/2019/04/018517, and JPRN-jRCTs051180081. We hope that our conclusions will be further confirmed after the successful completion of the above studies. In addition, some of the literature reports included in this study have shortcomings in the quality of methodology, such as unknown methods of randomization, unclear hidden distribution, and not using a blinded study design, as examples. In addition, this study has some incompleteness in obtaining literature data. For example, there are reports on the number of relapses within 1 year in the literature of Gellermann (11), but there are only the mean value and no standard deviation, without response through searching the original text or contacting the author. Thus, the study has to be excluded from the analysis of the outcome index. The authenticity of the research results may be affected. It is also suggested that when we carry out RCTs in the future, we should strictly abide by the above methodological requirements and report accordingly. In short, the treatment of mycophenolate mofetil in children with SDNS or FRNS still needs to be verified by more well-designed, large-sample, multicenter, long-term, and close follow-up RCTs.

## Data Availability Statement

The original contributions presented in the study are included in the article/supplementary material, further inquiries can be directed to the corresponding author/s.

## Author Contributions

XX was responsible for the concept, design, definition of intellectual content, literature search, data acquisition, and statistical analysis. S-YQ was responsible for the manuscript preparation and editing. MW took responsibility for the manuscript review and integrity of the work as a whole from inception to published article. All authors contributed to the article and approved the submitted version.

## Conflict of Interest

The authors declare that the research was conducted in the absence of any commercial or financial relationships that could be construed as a potential conflict of interest.

## Abbreviations

SDNS: steroid-dependent nephrotic syndrome
FRNS: frequently relapsing nephrotic syndrome
SRNS: steroid-resistant nephrotic syndrome
RNS: refractory nephrotic syndrome
SSNS: steroid-sensitive nephrotic syndrome
PNS: primary nephrotic syndrome.
